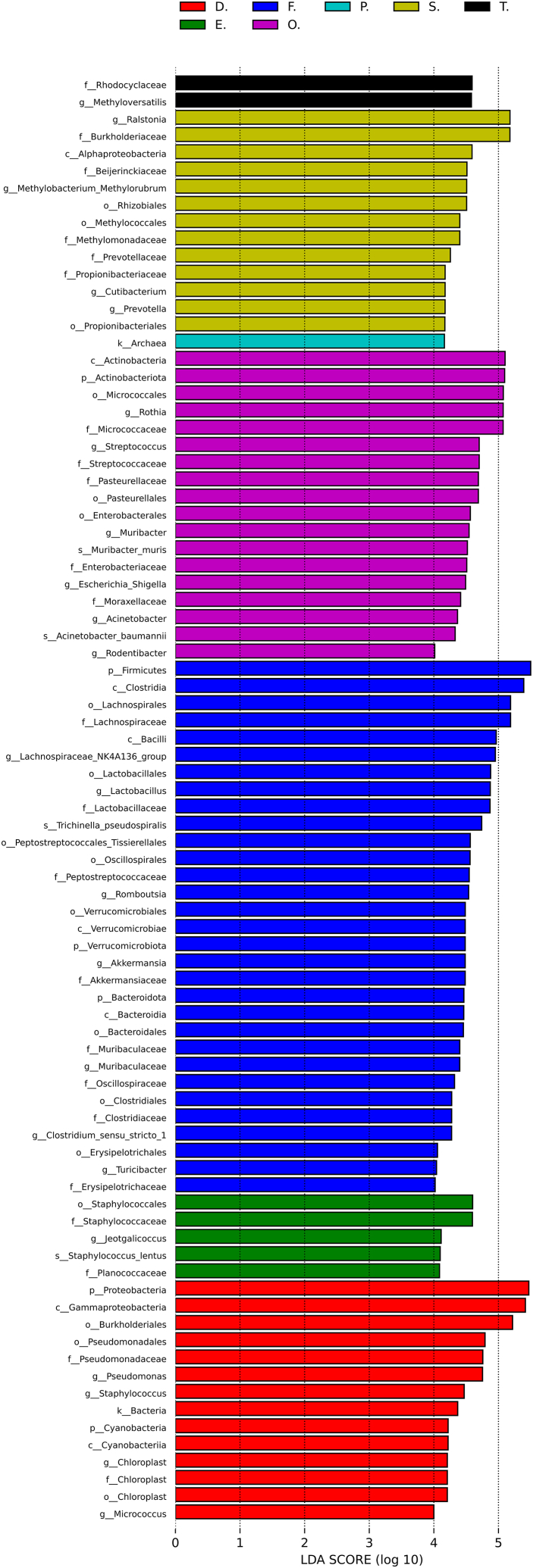# The microbiota continuum along the upper reproductive tract of male rat and its relation to semen parameters

**DOI:** 10.1016/j.heliyon.2024.e32556

**Published:** 2024-06-06

**Authors:** Guanjian Li, Qunshan Shen, Yang Gao, Cong Ma, Bing Song, Chao Wang, Dongdong Tang, Xiaojin He, Yunxia Cao

**Affiliations:** a Reproductive Medicine Center, the First Affiliated Hospital of Anhui Medical University, Hefei, Anhui, China; b National Health Commission Key Laboratory of Study on Abnormal Gametes and Reproductive Tract, Hefei, Anhui, China; c Key Laboratory of Population Health Across Life Cycle, Ministry of Education of the People's Republic of China, Hefei, Anhui, China; d Anhui Province Key Laboratory of Reproductive Health and Genetics, Hefei, Anhui, China; e Reproductive Medicine Center, Shanghai General Hospital, Shanghai Jiao Tong University School of Medicine, Shanghai, China

**Keywords:** Reproductive tract, Microbiota, Spermatogenesis, Sperm parameters, RNA sequencing

## Abstract

Given the physiological function and anatomical location of the reproductive tract, studying the upper reproductive tract microbiota may be essential for studying male infertility and other male diseases. This study aimed to characterize the microbiota of the upper reproductive tract male rats and investigate whether specific microbial compositions are associated with sperm parameters. 16S rRNA gene sequencing was used to characterize the microbial composition in the testis, epididymis, seminal vesicles, vas deferens and prostate tissues of the rats. The results showed significant enrichment of *Methyloperoxococcu*s spp. in testicular tissues, *Jeotgalicoccus* spp. in epididymal tissues. Spearman's correlation analysis revealed that the abundance of several bacterial genera in epididymal, testicular, and seminal vesicle gland tissues correlated with several sperm activity parameters. Our findings provide detailed information on characterizing the upper reproductive tract microbiome in male rats, as well as a potentially crucial link between the reproductive system microbiota and sperm quality.

## 1 Introduction

The collection of microorganisms on the body or surface of animals and humans comprises the internal microbiota [1]. High-throughput sequencing techniques in preclinical and clinical settings demonstrated that almost all tissues in the human body previously considered sterile contain microbes [2]. Moreover, microorganisms in the human body play critical roles in health and disease [3]. Microbiota dysbiosis can alter the immune response, host metabolism, gene expression patterns, and epigenetic modifications by affecting enzyme, hormone, and metabolite levels [4].

With the rapid development of high-throughput sequencing methods, microbial characterization can be completed quickly and efficiently in a culture-independent manner. The mouth, gut, skin, tumor tissue, and female reproductive tract have been extensively studied in human microbiome research [5,6]. However, the male upper reproductive tract has been largely overlooked. The reproductive tract microbiome is unique and can be influenced by various factors, such as sex, age, concomitant diseases, and sexual behavior [7]. However, few studies have focused on microorganisms in prostate cancer and testicular tissues [8,9]. Recently, bacterial DNA fingerprints were identified in testicular samples from men with idiopathic nonobstructive azoospermia, and dysbiosis of the bacterial microecology is associated with idiopathic nonobstructive azoospermia and complete germ cell aplasia [10].

Given the physiological function and anatomical location of the reproductive tract, studying the male upper reproductive tract microbiota may be essential for studying male infertility and other male diseases. Despite increasing literature reporting the microbiome composition in various male reproductive organ niches and clinical conditions [11], the bacterial types that are present in the upper reproductive tract, such as the epididymis and vas deferens, are unclear, and no detailed and comprehensive analyses of the male reproductive tract microbiome have been reported. Furthermore, the potential effect of the upper reproductive tract microbiome on sperm quality is not yet fully understood. Considering that the testes, epididymis, seminal vesicles, and prostate play vital roles in sperm development, maturation, storage, and expulsion, comprehensive testing of the microbiota in the reproductive system is necessary [12].

However, systematically identifying the “normal” upper reproductive tract microbiome has been challenging due to the difficulty of obtaining tissue in a disease-free state. Given ethical concerns, invasively collecting samples from healthy human volunteers is challenging, making it difficult to analyze the microbiota of the male upper reproductive tract directly and systematically. Therefore, investigating the characteristics of the microbiota in the upper reproductive tract samples of model animals may be of practical importance.

Another difficulty in assessing the presence of a normal upper reproductive tract microbiome is the constant threat of false-positive results owing to contamination. Culture-independent techniques for microbial detection typically involve ultrasensitive PCR-based assays, which are particularly vulnerable to contaminant DNA [13]. Therefore, rigorous control and eliminating contaminants are crucial for analyzing tissues with presumably low microbial biomass to obtain reliable data [14].

To characterize the microbiota in the samples, we used high-throughput 16S rRNA gene sequencing of testes, epididymal, seminal vesicles, vas deferens, ventral prostate, oral swabs, and cecum feces samples from rats. We aimed to determine whether microorganisms were present in the upper reproductive tract of male rats, whether there was continuity in the microbiome at each site, and whether specific microbial composition was associated with sperm parameters.

## 2 Materials and methods

### 2.1 Animals

Eight-week-old clean-grade male Sprague–Dawley (SD) rats were fed a control diet and provided free access to water. They were maintained on a 12-h light (8:00–20:00) and 12-h dark cycle. After one week of adaptive feeding, all male rats completed a mating experiment once a week thrice (excluding those unable to complete mating), and their upper reproductive tract microbiomes were analyzed at 12 weeks of age.

### 2.2 Sample collection

After euthanasia, oral swabs, cecal feces, and tissues from five upper reproductive tract organs: testicles, epididymis, vas deferens, seminal vesicle, and ventral prostate were collected from 12 male rats. Negative controls from the surface environment of the surgical equipment and laboratory reagents were collected and processed simultaneously with the tissue samples to control for possible microbial contamination. Sample collection was performed surgically in an air-purified operating room. After anesthesia by intraperitoneal injection of 0.8–0.9 mL of 1.2 % avertin into the male animals, the surgical area extending from the xiphoid process to the pelvic region was shaved of murine hair and then disinfected with a combination of betadine and 75 % alcohol swabs. All rats were not sexually active for 48 h. The samples of rat testis, epididymis, seminal vesicles, and prostate were processed in the biological safety cabinet in an ultra-clean room. Two experimenters sterilized their hands with medical alcohol gel and wore sterile surgical gowns, gloves and masks to collect all samples. The abdominal skin, muscles, and white lines of each animal were surgically incised. The testes, epididymis, prostate, and seminal vesicles were sequentially separated, excised, and placed in a sterile Petri dish. One side of the epididymis was cut into pieces and the spermatozoa within the epididymis were released into a sterile glass vial. Finally, oral swabs and cecal feces were collected using sterile nylon flocked cotton swabs. Five swab samples of the surface environment of the surgical equipment and laboratory reagents were also collected. The swab heads were then placed in sterile PBS. The mean surgical time after rat execution was 3.58 ± 0.67 min. All samples were placed on dry ice during the operation and then stored in a −80° refrigerator for subsequent DNA extraction.

### 2.3 Sperm motility assessment

Sperm from the epididymis were obtained and analyzed according to the World Health Organization (WHO) guidelines. Sperm analysis was performed following WHO guidelines. A CASA SCA microscope (5.4, Microptic SL, Barcelona, Spain) was used to analyze sperm concentration and motility [15]. Sperm samples were collected from the distal caput and cauda of the epididymis and used for computer-assisted sperm analysis (CASA) with reference to the methods used in previous studies [16,17].

In brief, the epididymis was trimmed of fat and clamped at the caput-corpus and corpus-cauda junctions, then severed and blotted on the corpus side of the clamp. The caput and cauda epididymides were rinsed in separate 35-mm Petri dishes containing 2 ml of motility media and then transferred to separate 35-mm Petri dishes containing 3 ml of fresh media while still clamped. Several tubules in the distal caput and cauda regions were pierced with a scalpel blade, allowing sperm to diffuse into the medium. After removing the tissue, the sperm were allowed to disperse for about 5 min. The following sperm motility parameters were measured: average path velocity (VAP; l m/s), the amplitude of lateral head displacement (ALH), curvilinear velocity (VCL; l m/s), straight-line velocity (VSL; l m/s), beat cross frequency (Hz), linearity (%), and straightness (%).

### 2.4 DNA extraction and 16S rRNA gene sequencing

We characterized the microbiota in the samples through 16S rRNA gene analysis. For intestinal contents samples, DNA was extracted by using Stool DNA Kit (TianGen, China, Catalog #: DP712). For other types of samples, DNA was extracted by using CTAB extraction method. The daughter 16S rRNA genes (16SV3–V4) were amplified using specific primers with barcodes. The primers used were: 5′CCTACGGGNGGCWGCAG3’ (forward primer) and 5′GACTACHVGG GTATCTAATCC3’ (reverse primer). All PCR reactions were carried out with 15 μL of Phusion® High Fidelity PCR Master Mix (New England Biolabs); 2 μM of forward and reverse primers, and about 10 ng template DNA. Thermal cycling consisted of initial denaturation at 98 °C for 1 min, followed by 30 cycles of denaturation at 98 °C for 10 s, annealing at 50 °C for 30 s, and elongation at 72 °C for 30 s and 72 °C for 5 min. Mix same volume of 1X loading buffer (contained SYB green) with PCR products and operate electrophoresis on 2 % agarose gel for detection. PCR products were mixed in equidensity ratios. Then, mixture PCR products were purified with Universal DNA Purification Kit (TianGen, China, Catalog #: DP214). libraries were generated using NEB Next® Ultra™ II FS DNA PCR-free Library Prep Kit (New England Biolabs, USA, Catalog #: E7430L) following manufacturer's recommendations and indexes were added.

The library was checked with Qubit and real-time PCR for quantification and bioanalyzer for size distribution detection. Quantified libraries were pooled and sequenced on Illumina platforms, according to effective library concentration and data amount required.

### 2.5 Bioinformatics, and statistical analyses

Paired-end reads was assigned to samples based on their unique barcode and truncated by cutting off the barcode and primer sequence. Paired-end reads were merged using FLASH (V1.2.11, http://ccb.jhu.edu/software/FLASH/), a very fast and accurate analysis tool, which was designed to merge paired-end reads when at least some of the reads overlap the read generated from the opposite end of the same DNA fragment, and the splicing sequences were called raw tags. Quality filtering on the raw tags were performed using the fastp (Version 0.23.1) software to obtain high-quality Clean Tags. The tags were compared with the reference database (Silva database (16S/18S), https://www.arb-silva.de/; Unite Database (ITS), https://unite.ut.ee/) using UCHIME Algorithm (http://www.drive5.com/usearch/manual/uchime_algo.html) to detect chimera sequences, and then the chimera sequences were removed. Then the effective tags were finally obtained. For the Effective Tags obtained previously, denoise was performed with DADA2 or deblur module in the QIIME2 software (Version QIIME2-202006) to obtain initial ASVs (Amplicon Sequence Variants) (default: DADA2), and then ASVs with abundance less than 5 were filteredout. Species annotation was performed using QIIME2 software, the annotation database is Silva Database. The absolute abundance of ASVs was normalized using a standard of sequencenumber corresponding to the sample with the least sequences. Subsequent analysis of alpha diversity and beta diversity were all performed based on the output normalized data. In order to analyze the diversity, richness and uniformity of the communities in the sample, alpha diversity was calculated from 2 indices in QIIME2, including Shannon and Simpson. Shannon (http://scikit-bio.org/docs/latest/generated/skbio.diversity.alph a.shannon.html#skbio.diversity.alpha.shannon); Simpson (http://scikit-bio.org/docs/latest/generated/skbio.diversity.alph a.simpson.html#skbio.diversity.alpha.simpson); In order to evaluate the complexity of the community composition and compare the differences between samples(groups), beta diversity was calculated based on weighted unifrac distances in QIIME2.

Cluster analysis was performed with principal component analysis (PCA), which was applied to reduce the dimension of the original variables using the ade4 package and ggplot2 package in R software (Version 3.5.3).

Principal Coordinate Analysis (PCoA) was performed to obtain principal coordinates and visualize differences of samples in complex multi-dimensional data. A matrix of weighted unifrac distances among samples obtained previously was transformed into a new set of orthogonal axes, where the maximum variation factor was demonstrated by the first principal coordinate, and the second maximum variation factor was demonstrated by the second principal coordinate, and so on. The two-dimensional PCoA results were displayed using ade4 package and ggplot2 package in R software (Version 2.15.3). The Non-Metric Multi-Dimensional Scaling (NMDS) analysis was performed using the vegan package in R software v3.3. The LEfSe software (Version 1.0) was used to do LEfSe analysis (LDA score threshold: 4) so as to find out the biomarkers.

Further, to study the functions of the communities in the samples and find out the different functions of the communities in the different groups, the PICRUSt2 software (Version 2.1.2-b) was used for function annotation analysis. The Kyoto Encyclopedia of Genes and Genomes (KEGG) pathway terms found by this method were then correlated with the relative abundance of the select bacterial species.

Spearman's rank order correlation was used to find associations between the parameters. All calculations were performed using SPSS statistical software (version 23, IBM SPSS, Chicago, IL, USA). Probability values of less than 0.05 were considered significant.

## 3 Results

### 3.1 Sample collection and 16S rRNA sequencing

We characterized the microbiota of 12 male SD rats (3-month-old) mated thrice with females. Testes, epididymal, seminal vesicles, vas deferens, ventral prostate, oral swabs, and cecum feces samples were collected from each rat. Finally, 83 samples were obtained for microbiota analysis (one oral swab sample was not tested because of accidental contamination during transportation). Five swab samples were collected from the surface environment of the surgical equipment and laboratory reagents.

**Fig. 1:**
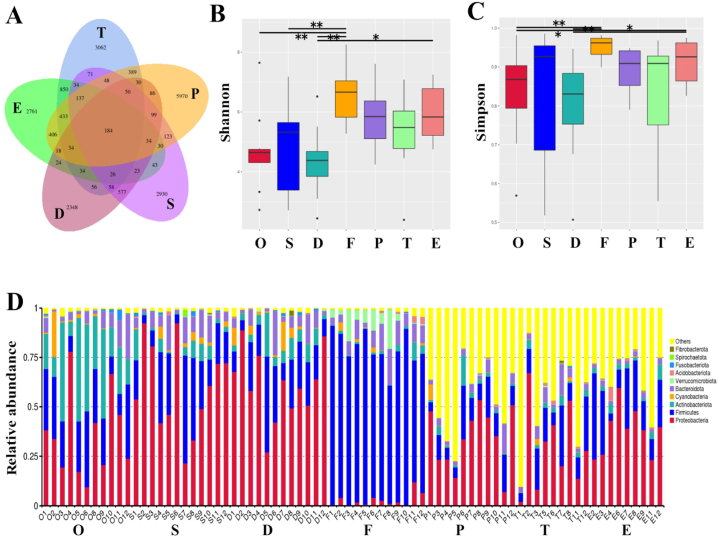
Taxonomic classification and microbial diversity of samples. (A) Venn diagram demonstrating the overall overlap of amplicon sequence variants (ASVs) according to the sample source. (B, C) Alpha-diversity indices of the Shannon and Simpson indices of microbiota based on the diversity of microbial communities. (D) The component proportion of bacterial phylum (top 10) in the samples. *P < 0.05, **P < 0.01. O, oral swab; S, seminal vesicle; D, vas deferens; F, cecum feces; P, ventral prostate; T, testicles; E, epididymis.

Of the 88 samples, 11 oral swabs, 12 seminal vesicles, 12 vas deferens, 12 cecum stools, 11 ventral prostate, nine testicular, and nine epididymal samples contained detectable 16S rRNA levels. However, 16S rRNA could not be amplified in one prostate, three testicular, and three epididymal samples, implying that the testicular, epididymal, and prostate tissues of some male rats may contain no or very low levels of bacteria. In addition, all five background control swabs were excluded from further analysis because they did not show DNA amplification or zero reads were obtained after sequencing. After sequence assembly, quality filtering, and data processing, 20897 unique ASVs were identified in all the reproductive tract samples. Of these, 3062 ASVs (14.7 %) were unique to the testes, 2761 (13.2 %) to the epididymis, 2348 (11.2 %) to the vas deferens, 5970 ASVs (28.57 %) to the prostate, and 2930 (14.0 % of all 14.0 %) to the seminal vesicles (Fig. 1A).

### 3.2 Species abundance and diversity analysis

Phylum-, family-, and genus-level analyses identified 59 bacterial phyla, 669 families, and 1681 genera. Alpha-diversity analysis (Shannon and Simpson indices) revealed that cecum fecal samples had significantly higher species richness than the upper reproductive tract samples. In contrast, vas deferens specimens had the lowest species diversity among all samples (Fig. 1B and C). The top five bacterial phyla (mean relative abundance) in all reproductive tract samples were Proteobacteria, Firmicutes, Actinobacteria, Cyanobacteria, and Bacteroidetes, whereas Firmicutes and Actinobacteria were predominant in the fecal and oral swabs (Fig. 1D). At the phylum level, Firmicutes dominated the fecal microbiota, compared to large proportions of Proteobacteria, Actinobacteria, and Cyanobacteria in the upper reproductive tract. The most abundant bacterial genera in the upper reproductive tract samples included *Ralstonia*, *Pseudomonas*, *Staphylococcus*, *Shigella*, and *Jeotgalicoccus*. However, the top ten bacterial genera regarding mean relative abundance varied among the sample types (Table 1).

**Table 1:** Top 10 bacterial genera regarding mean relative abundance in upper reproductive tract tissue samples.

	Testicles	Epididymis	Vas deferens	Seminal vesicle	Prostate
1	*Pseudomonas*	*Ralstonia*	*Ralstonia*	*Ralstonia*	*Ralstonia*
2	*Methyloversatilis*	*Pseudomonas*	*Pseudomonas*	*Methylobacterium*	*Pseudomonas*
3	*Ralstonia*	*Staphylococcus*	*Methylobacterium*	*Pseudomonas*	*Escherichia*
4	*Sphingomonas*	*Escherichia*	*Staphylococcus*	*Methylosarcina*	*Megamonas*
5	*Prevotella*	*Jeotgalicoccus*	*Methylosarcina*	*Cutibacterium*	*Cutibacterium*
6	*Staphylococcus*	*Acinetobacter*	*Streptococcus*	*Prevotella*	*Muribaculaceae*
7	*Lactobacillus*	*Clostridium*	*Methyloversatilis*	*Enhydrobacter*	*Clostridium_sensu*
8	*Lysinibacillus*	*Lysinibacillus*	*Chloroplast*	*Streptococcus*	*Prevotella*
9	*Acinetobacter*	*Streptococcus*	*Cutibacterium*	*Staphylococcus*	*Mitochondria*
10	*Bacteroides*	*Lactobacillus*	*Prevotella*	*Chloroplast*	*Stenotrophomonas*

**Fig. 2:**
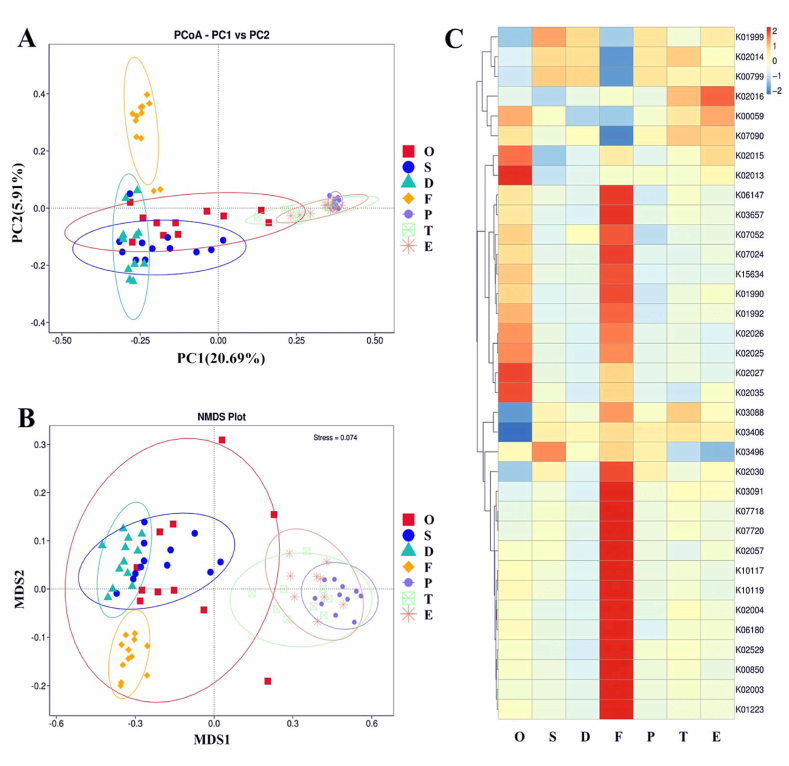
Species composition differences and functional prediction analysis among sample groups. (A) β-diversity indices of principal coordinate analysis (PCoA) plots of microbiota based on weighted UniFrac distances among the samples. (B) Non-metric multidimensional scaling (NMDS) analysis shows between-group differences in the samples. (C) Functional prediction based on the Kyoto Encyclopedia of Genes and Genomes database was performed, and heat maps were created using the top 35 functions regarding abundance and their abundance information. O, oral swab; S, seminal vesicle; D, vas deferens; F, cecum feces; P, ventral prostate; T, testicles; E, epididymis.

Beta-diversity analysis clustered upper reproductive tract samples roughly into two microbiota profiles, with specimens from testicular, epididymal, and prostate tissues having similar bacterial compositions to each other. Those from seminal vesicles and vas deferens tissues had a similar bacterial composition. The two sets of reproductive tract samples were separated using principal coordinate analysis (PCoA) based on the weighted UniFrac distance (Fig. 2A). Similar to the PCoA analysis, non-metric multidimensional scaling analysis results completely separated these two specimen groups and the cecum stool. Some similarities in the bacterial composition of oral swabs and upper reproductive tract samples were observed, especially in the seminal vesicles and vas deferens (Fig. 2B).

### 3.3 Linear discriminant analysis effect size

Linear discriminant analysis effect size (LEfSe) analysis was used to investigate bacterial biomarkers across groups, and discriminatory taxa with linear discriminant analysis scores greater than four were selected (Supplemental Fig. 1). The genera *Methyloversatilis* and *Jeotgalicoccus* were significantly enriched in testicular and epididymal tissues, respectively. The microbiota of the seminal vesicle tissue was characterized by the predominance of the genera *Ralstonia*, *Prevotella*, and *Cutibacterium*, whereas the signature bacterial genera in the vas deferens samples included *Pseudomonas*, *Staphylococcus*, *Chlorobacterium*, and *Micrococcus*.

### 3.4 Functional prediction analysis

The Kyoto Encyclopedia of Genes and Genomes database was used to annotate genes in the gene catalog. The testes and epididymis samples showed a higher proportion of genes involved in the anabolism of substrate-binding proteins, membrane proteins, membrane receptor proteins, and carrier proteins of the transport system, whereas genes involved in branched-chain amino acid transport and glutathione transport were enriched in the prostate and vas deferens samples. Spermatophore gland samples were enriched in chromosome-partitioning proteins and branched-chain amino acid transport system-related proteins (Fig. 2C).

### 3.5 Sperm parameters and correlation analysis

**Fig. 3:**
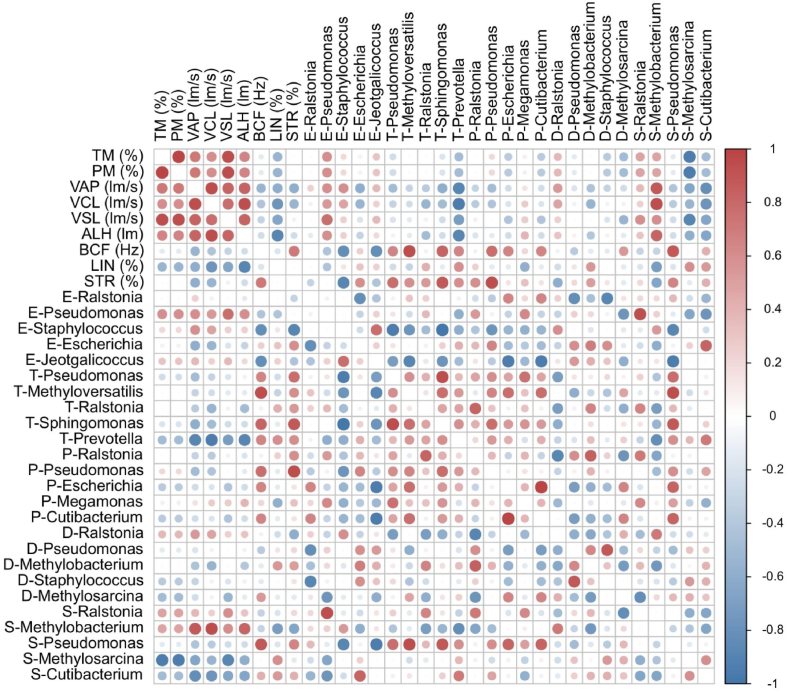
Heat map of Spearman's rank correlation coefficients between the top five bacterial genera at each sample site and epididymal sperm parameters. TM, total motility; PM, progressive motility; VAP, average path velocity; VCL, curvilinear velocity; VSL, straight-line velocity; ALH, lateral head displacement; BCF, beat cross frequency; LIN, linearity; STR, straightness.

We used Spearman's rank correlation test to investigate the relationship between the microbiome (the top five bacterial genera at each sample site) and sperm parameters in the upper reproductive tract samples. The sperm motility parameters for all rats are shown in Table 2. Spearman's correlation analysis between the two variables revealed that the abundances of many bacterial genera were interrelated. Furthermore, certain bacterial genera were associated with sperm motility (Fig. 3). The abundance of *Pseudomonas* in the epididymis positively correlated with sperm parameters such as total motility, progressive motility, VSL, and ALH. In contrast, the abundance of *Prevotella* in the testes negatively correlated with sperm motility parameters such as VAP, VCL, and ALH. Notably, the abundances of the genera *Chloroplast*, *Staphylococcus*, and *Methylosarcina* in seminal vesicles were also related to sperm activity parameters.

**Table 2:** Epididymal sperm parameters provided by a computer-assisted sperm analysis system.

	S1	S2	S3	S4	S5	S6	S7	S8	S9	S10	S11	S12
TM (%)	29.2	57.6	101.4	88.2	66.2	78.6	36.4	30.8	53.8	73.8	20	31.2
PM (%)	6.6	15.2	46.0	41.6	23.6	28.2	14.8	7.0	15.4	28.4	4.6	8.4
VAP (lm/s)	110.85	115.10	155.81	153.69	122.21	128.73	100.98	97.17	107.07	127.77	109.25	111.39
VCL (lm/s)	221.98	216.48	313.36	315.20	240.44	273.06	196.04	196.82	208.56	257.46	206.90	223.38
VSL (lm/s)	84.36	84.615	122.25	125.19	96.165	99.45	83.67	79.29	83.94	96.84	73.68	84.99
ALH (lm)	16.26	15.44	20.48	20.88	17.30	18.14	13.06	13.66	14.08	17.72	13.56	15.82
BCF (Hz)	17.64	18.06	21.16	16.15	19.18	31.37	29.31	42.56	23.76	18.82	23.14	16.38
LIN (%)	54.23	53.44	52.29	53.77	53.70	51.22	59.38	54.84	56.86	54.35	56.03	55.93
STR (%)	80.78	77.12	76.44	81.68	77.41	78.97	79.70	80.44	79.58	78.23	71.02	78.15

TM, total motility; PM, progressive motility; VAP, average path velocity; VCL, curvilinear velocity; VSL, straight-line velocity; ALH, lateral head displacement; BCF, beat cross frequency; LIN, linearity; STR, straightness.

## 4 Discussion

We systematically obtained tissue samples from the upper reproductive tract of sexually mature rats and characterized their microbiomes using 16S rRNA gene sequencing. The presence of distinct bacterial communities throughout the upper reproductive tract of male rats was observed, forming a continuum of microbiota in the testes, epididymis, vas deferens, seminal vesicle glands, and prostate. As expected, many organisms in the testes and epididymis tissues were also in the prostate, seminal vesicles, and vas deferens. Samples from the testicular, epididymal, and prostate tissues had a similar bacterial composition, whereas samples from the seminal vesicle and vas deferens tissues had a more similar bacterial composition.

Because of its topography, the urethra and upper reproductive tract may be reached by skin and gut microbiota members, which can survive within the specific reproductive tract microenvironment and outcompete other microbes. Nutrient availability, osmotic pressure, adhesion sites, and immune interactions are also associated with microbiome formation [18]. In addition, the pH of the upper reproductive tract and oxygen availability may play roles in forming specific microbiomes [19]. Acute and chronic orchitis, epididymitis, seminal vesiculitis, and prostatitis are common in clinical practice, suggesting that the upper genital tract may be invaded by uropathogens and sexually transmitted bacteria, despite the presence of immune mechanisms similar to BTB and the immune arsenal [20].

The genera *Blautia*, *Clostridium*, and *Prevotella* were found in the testicular specimens of infertile men [9]. Furthermore, when analyzing human sperm samples, *Prevotella* was associated with low-quality sperm [21,22]. *Prevotella* was also present in most testicular samples in the current study, and its abundance was negatively correlated with sperm motility indicators (such as VAP, VCL, and ALH). This finding supports the possibility that upper reproductive tract microbes contribute to the downstream seminal microbiome composition and implies that *Prevotella* spp. may cause spermatogenesis defects and male infertility [23].

The epididymis is less immunoprivileged than the testes, with some degree of immunoreactivity in the sperm head and inflammatory features in its tail [24]. Due to the scarcity of human epididymal specimens, studies on epididymal microorganisms have focused on rodent tissue samples. In antibiotic-treated animals, substantial differences in epididymal ultrastructure and purine metabolism have been observed [25]. Acute and chronic epididymitis are common male reproductive tract infections, and persistent pathogen damage leads to fibrotic transformation and epithelial degeneration of the epididymis [26]. Patients with epididymitis have poor semen quality and changes in the protein composition of their spermatozoa, leading to male infertility [27,28].

The seminal vesicles are pairs of tubular glands with the lower part connected to the end of the ipsilateral vas deferens [29]. Seminal vesicles secrete fluid, the primary component of the sperm. Our current and previous studies provide direct evidence that a resident microbiome may be present in human and animal seminal vesicles [30]. The most abundant bacterial genera in the seminal vesicles included *Ralstonia*, *Methylobacterium*, *Pseudomonas*, *Methylosarcina*, and *Prevotella*. Infection and inflammation in this area may lead to infertility due to decreased semen quality, hematospermia, and ejaculatory duct obstruction [31].

These findings suggest that the seminal vesicles, epididymis, and testicles of humans and animals may have long-term symbiotic relationships with microorganisms, including potentially widespread atypical and subclinical infections. The impact of localized microbial pathogens in the reproductive system on spermatogenesis and semen quality may be far greater than previously thought.

In our study, multiple bacterial genera in the rat epididymis, testes, and seminal vesicles correlated with several sperm motility parameters. The microbiota may influence the environment in which spermatogenesis and maturation occur, thus affecting sperm physiology. Although testicular microbiota have been detected in some studies, their role in testes spermatogenesis remains controversial [32]. The testicular microbiome appears to expand interleukin 17, producing gamma-delta T cells during puberty and promoting gonadal immune surveillance [33]. Furthermore, bacterial translocation-mediated pathogen response can cause endothelial damage, BTB subversion, and spermatogenesis and viability alteration [34]. Dendritic cells and macrophage-infiltrating tissues capture the spermatozoa, impairing spermatogenesis [35]. From a systemic perspective, the microbiome promotes homeostasis in the body and aids immune system development [36,37]. A systemic inflammatory state can be induced during dysbiosis, potentially harming the male reproductive system.

The upper reproductive microbiota is related to the gut microbiota, the largest and most diverse community of microbes in the human body [38]. Both types of microbiota may play roles in immune system development and pathogen defense. Concurrent pathologies, such as infections, drugs, obesity, diabetes, and trauma, can cause microbial dysbiosis [39]. Microecological interventions such as dietary interventions, probiotics, prebiotics, antimicrobials, and antioxidants may restore otherwise impaired male reproductive potential. Notably, microbial immunotherapy and targeted therapy for improving male fertility can easily overcome the limitations of conventional therapies and should be based on further in-depth studies of the gut and genitourinary microbiomes.

The current findings revealed the microbial composition of the upper reproductive tract. However, the upper reproductive tract appears to be a low microbial biomass site. Therefore, microbiome analysis of sites with low microbial biomass requires special attention to microbial contamination of the environment and laboratory reagents to determine the true bacterial sequence [40]. The current study used strictly sterile sample collection methods, sterile reagents, and additional background control samples were collected for analysis. Ultimately, none of the background control swabs showed DNA amplification or yielded zero read lengths after sequencing, suggesting that surface contamination of the samples during sampling or reagent contamination during testing is unlikely to be the primary source of these microorganisms. Therefore, the potential contamination step for microbiome analysis was well controlled, and we obtained reliable microbiome data from the upper reproductive tract of rats.

It is noteworthy that considering the bacterial composition of the urogenital tract could be influenced by factors such as age and sexual activity, using sexually mature, mated male rats as a model for studying the microbiome of the male upper reproductive tract may better correspond to our target population: sexually active adult males facing fertility issues. Indeed, studies on the human semen microbiome have shown that men with sexual experience have a higher total bacterial abundance and microbial diversity compared to men without sexual experience (p < 0.05) [41]. Additionally, there is evidence suggesting semen and vaginal microbiomes of sexual partners may be similar [42]. Sexual intercourse can lead to bacterial exchange between the reproductive tracts of partners, which could influence fertilization [[43], [44], [45]].

The main strength of this analysis lies in the novel systematic characterization of the microbiome environment associated with the upper reproductive tract of male rats, highlighting the possible contribution of reproductive tract microbes in spermatogenesis and maturation. Our results call for further investigation of microbial colonization and pathophysiological roles in the male urogenital tract and, as with the female reproductive tract, open potential niches for therapeutic avenues of microecological intervention.

However, this study had some limitations. First, although our data provide evidence for the presence of bacteria and bacterial DNA in the upper reproductive tract of rats, the viability of these bacteria remains unclear. Microbiome research confronts technical and methodological challenges, as detection and analytical techniques are far from standardized. Understanding the intricate host-microbiome interactions, along with the confounding effects of various physiological and environmental factors, poses an additional challenge. Second, this preliminary study could not assess the causality between microorganisms and semen quality. Furthermore, the currently reported sperm parameters are from epididymal sperm analyses, how the microbiome in the seminal vesicles influences epididymal sperm parameters may be complex, and the representativeness of epididymal sperm parameters on the “final” semen quality also needs to be further evaluated. Additionally, animals and humans differ, and the reproductive tract flora of rats may not reflect the true state of the human reproductive tract flora. However, despite these limitations, such as the potential for contamination and the lack of further functional validation, the current study systematically characterized the microbial microenvironment of the upper reproductive tract, an interesting and novel finding as it provides the opportunity to consider the issue of male reproductive potential and its future management from a different perspective.

## 5 Conclusion

In conclusion, this study provided novel detailed data on various organs of the upper reproductive tract of rats with unique microbial characteristics. Our study suggests that a progressively changing microbial continuum exists within individuals, from the testes to the prostate. Our findings also imply a potentially essential link between the upper reproductive system microbiota and the development of sperm function and provide a proof of principle for future human studies in this area. However, further species-level studies are needed to assess microbial action mechanisms in spermatogenesis and explore microbiota-targeted therapeutic strategies to address potential male infertility.

## Data availability statement

All data and methods necessary to reproduce this study are included in the manuscript. Sequencing data were deposited to the National Center for Biotechnology Information under accession number SUB13920575 are publicly available. This research does not report original code. Other original data reported in this paper will be shared by the lead contact upon request.

## Ethics approval statement

The experimental protocol was approved by the Biomedical Ethics Committee of Anhui Medical University (approval number: 80181215) and was conducted following the recommendations of the Guide for the Care and Use of Laboratory Animals of the National Institutes of Health.

## CRediT authorship contribution statement

**Guanjian Li:** Writing – review & editing, Writing – original draft, Conceptualization. **Qunshan Shen:** Writing – review & editing, Writing – original draft, Supervision. **Yang Gao:** Writing – review & editing, Writing – original draft, Supervision. **Cong Ma:** Writing – review & editing, Writing – original draft, Supervision. **Bing Song:** Writing – review & editing, Writing – original draft, Supervision. **Chao Wang:** Writing – review & editing, Writing – original draft, Supervision. **Dongdong Tang:** Writing – review & editing, Writing – original draft, Supervision. **Xiaojin He:** Writing – review & editing, Supervision, Formal analysis. **Yunxia Cao:** Writing – review & editing, Supervision, Formal analysis.

## Declaration of competing interest

The authors declare that they have no known competing financial interests or personal relationships that could have appeared to influence the work reported in this paper.

## Figures and Tables

**figs1:**